# A multi-Fc-species system for recombinant antibody production

**DOI:** 10.1186/1472-6750-9-14

**Published:** 2009-02-26

**Authors:** Sandrine Moutel, Ahmed El Marjou, Ole Vielemeyer, Clément Nizak, Philippe Benaroch, Stefan Dübel, Franck Perez

**Affiliations:** 1 Translational Research Department, 26 rue d'Ulm, F75248 Paris Cedex 05, France; 2 CNRS UMR144, 26 rue d'Ulm, F75248 Paris Cedex 05, France; 3 INSERM U653, 26 rue d'Ulm, F75248 Paris Cedex 05, France; 4 Research Center, Institut Curie, 26 rue d'Ulm, F75248 Paris Cedex 05, France; 5 CNRS, UMR 5588, Université Joseph Fourier -, BP 87, 140 avenue de la Physique, Domaine Universitaire, 38402 Saint Martin d'Hères Cedex, France; 6 Dept. Biotechnology Technical, University of Braunschweig, Spielmannstr. 7, 38106 Braunschweig, Germany

## Abstract

**Background:**

Genomic, transcriptomic and proteomic projects often suffer from a lack of functional validation creating a strong demand for specific and versatile antibodies. Antibody phage display represents an attractive approach to select rapidly *in vitro *the equivalent of monoclonal antibodies, like single chain Fv antibodies, in an inexpensive and animal free way. However, so far, recombinant antibodies have not managed to impose themselves as efficient alternatives to natural antibodies.

**Results:**

We developed a series of vectors that allow one to easily fuse single chain Fv antibodies to Fc domains of immunoglobulins, improving their sensitivity and facilitating their use. This series enables the fusion of single chain Fv antibodies with human, mouse or rabbit Fc so that a given antibody is no longer restricted to a particular species. This opens up unlimited multiplexing possibilities and gives additional value to recombinant antibodies. We also show that this multi-Fc species production system can be applied to natural monoclonal antibodies cloned as single chain Fv antibodies and we converted the widely used 9E10 mouse anti-Myc-tag antibody into a human and a rabbit antibody.

**Conclusion:**

Altogether, this new expression system, that brings constant quality, sensitivity and unique versatility, will be important to broaden the use of recombinant and natural monoclonal antibodies both for laboratory and diagnosis use.

## Background

Antibodies are essential tools for the identification and study of proteins involved in normal and pathological functions. Our need for specific antibodies will further increase in the post-genomic era [1]. Recombinant antibodies like single chain Fv (scFv) represent an attractive alternative to natural antibodies. In particular, they can be selected using synthetic *in vitro *approaches like phage or ribosome display allowing fast, specific, animal-experiment independent and rather inexpensive selection of antibody [2]. These antibodies can then be used, in principle, in any approach where natural antibodies are usually employed.

Nevertheless, this method of antibody generation has not imposed itself within academic use and almost no such recombinant antibodies are distributed commercially as laboratory or diagnosis reagents. This is rather surprising as currently available libraries are of large enough diversity to provide a high success rate with a very low technological investment. Some large scale approaches are currently developed partly based on recombinant antibodies [[3,4]; see also ] and we and others even showed that this approach allows the selection of antibodies that would be hard/impossible to obtain by other means [see for example [5-7]]. One of the main reasons for this lack of popularity is probably the general feeling that the sensitivity of recombinant antibodies is lower than that of natural antibodies. The apparent reduced affinity is mostly due to the fact that scFv are monovalent molecules that lack the avidity binding obtained through dimerization. Another limitation is that the end product is not exactly an antibody, but only an antibody fragment, which is more complicated to use than its natural counterpart.

To solve these limitations, we developed a series of expression vectors based on the pFuse expression system (commercially available from InvivoGen, see materials and methods) that allow expression of scFv in fusion with natural Fc regions. This approach strongly improved antibody sensitivity and ease of use, and additionally provided so far unavailable versatility since scFv can be fused to human, mouse and rabbit Fc in an easy one step cloning procedure. We further showed that this method can be applied to natural antibodies re-cloned as scFv. Thus, we fused the monoclonal anti-Myc antibody 9E10 to human and rabbit Fc and showed that, as for recombinant antibodies, it provides extended multiplexing possibilities. We believe that the described method will be decisive in allowing the recombinant antibody approach to impose itself as a robust and powerful alternative option for antibody isolation and usage.

## Results

### Plasmids construction and antibody production

**Figure 1:**
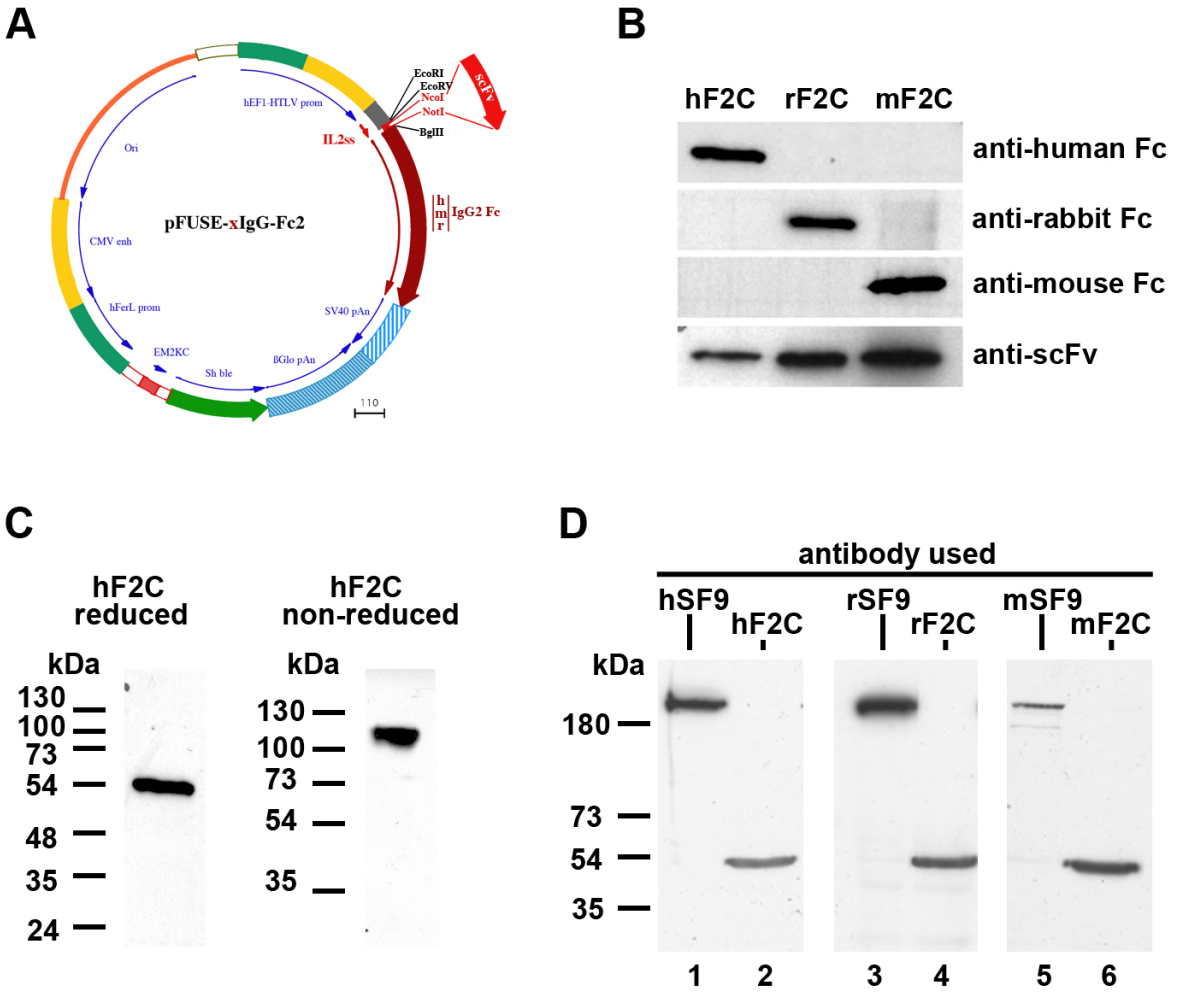
**Production and use of multi-species scFv-Fc antibodies**. **A: **Schematic map of the generic pFuse-xIgG-Fc2 used to express scFvs (adapted from Invivogen). Three vectors are available bearing either a human (pFuse-hIgG-Fc2), mouse (pFuse-mIgG-Fc2) or rabbit IgG2 (pFuse-rIgG-Fc2). The scFv is inserted (NcoI/NotI) inframe between the IL2 secretion signal and the Fc portion. The vector can be selected using Zeocin both in *E. coli *and in mammalian cells. **B: **Western Blot analysis of the scFv F2C fused to Fc domains from 3 different species. hF2C, rF2C and mF2C secreted by CHO cells were purified on protein A and analyzed by western blot using either species-specific anti-Fc antibody, or using a polyclonal anti-scFv. Each F2C fusion antibody is detected in CHO supernatants and is respectively seen as a human, rabbit or mouse antibody.**C**: scFv-hFc are expressed as dimers. hF2C proteins were separated by SDS-PAGE either in reducing (left) or in non-reducing conditions (right) and analyzed by western blot. This confirmed that hF2C behaves as a dimer in non-reducing conditions. **D**: Using multi-species scFv-Fc antibodies for Western blot immuno-labeling. A HeLa cell protein lysate was separated by SDS-PAGE and transferred on a nitrocellulose membrane. The membrane was probed using anti-MyosinII SF9 (lane 1, 3 and 5) or anti-Tubulin F2C (2, 4, 6) antibodies fused to either human (h; 1,2), rabbit (r; 3,4) or mouse (m; 5,6) Fc. This experiment shows that each scFv kept its specificity when used as a human, mouse or rabbit antibody.

Our plasmids are based on the pFUSE-Fc2(IL2ss)™ series from Invivogen (San Diego, USA) that contains the interleukin-2 (IL2) signal sequence and allows the secretion of Fc-Fusion proteins by mammalian cells. They are selectable using Zeocin™ (Zeo) both in prokaryotic and eukaryotic cells. These plasmids were modified by site directed mutagenesis and adaptor insertion (see Material and Methods, Figure 1A) to allow the easy one step cassette cloning of recombinant antibodies extracted from a large collection of common recombinant antibody selection and expression plasmids (e.g pHEN, pSEX, pHAL, pCANTAB, pHOG, pOPE, pSTE). Three plasmids were constructed enabling fusion of scFv at their C-terminus with either human IgG2 (h), mouse IgG2a (m) or the rabbit IgG (r) Fc domain (Fc regions comprise the CH2 and CH3 domains of the IgG heavy chain and the hinge region).

We sub-cloned the F2C antibody directed against alpha-Tubulin [8] in fusion with the human, mouse or rabbit Fc domains generating antibodies hF2C, mF2C and rF2C, respectively. Three days after transient transfection of CHO cells with these expression plasmids, secreted antibodies were purified from cell supernatants using proteinA-affarose and loaded on SDS-PAGE. Western blotting analysis showed that each CHO supernatant expressed the expected 53 kDa antibody that can be specifically detected using anti-IgG antibodies directed against the respective species (Figure 1B). Because the scFvs were fused to the hinge domain of IgGs, the Fc domains were expected to form di-sulfide bridges. We confirmed using SDS-PAGE analysis that, in non-reducing conditions, the scFv-Fc behave as dimeric proteins while they migrate as monomers upon reduction (Figure 1C).

### scFv versus scFv-hFc as tools for western blotting and immunofluorescence

We tested whether scFv-Fc could be efficiently used in western blotting experiments. NP40 solubilised lysates of HeLa cells were separated by SDS-PAGE and transferred to nitrocellulose membrane. The membrane was cut and incubated with either F2C or SF9 (directed against nonmuscle MyosinIIA [8]) fused to either human, mouse or rabbit Fc domains. The membrane strips were then incubated with species-specific HRP-labeled secondary antibodies and subsequently developed. As shown in Figure 1D, we found that a specific band was detected in each condition indicating that direct dilution of unpurified culture medium from transfected CHO cells transiently expressing scFv-xFc (where x means human, rabbit or mouse) can be used for western blot experiments.

**Figure 2:**
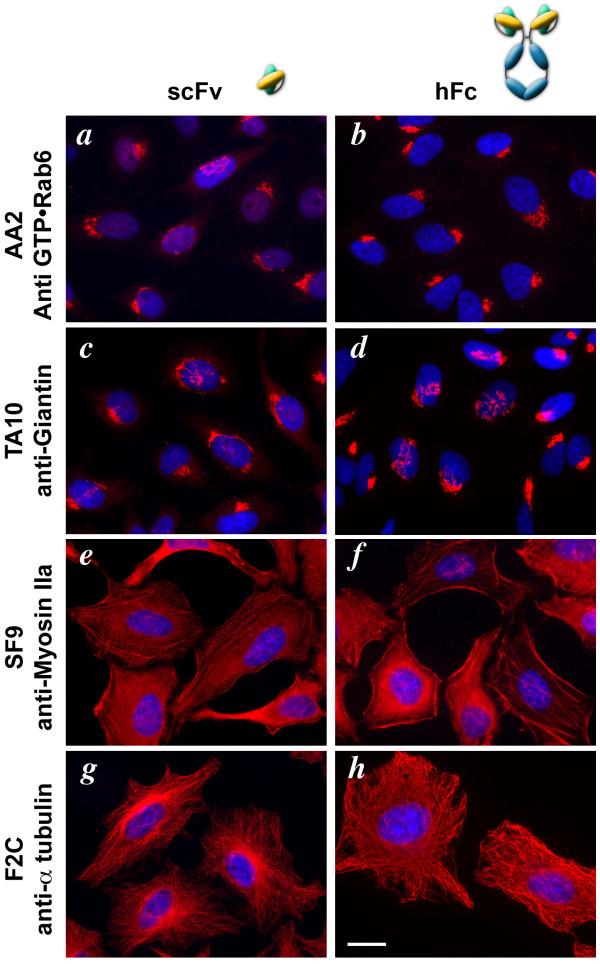
**Using scFv-Fc antibody for immunofluorescence**. Cells were immuno-labeled using either scFv (a, c, e, g) or scFv-hFc (b, d f, h) antibodies. scFv were detected using an anti-Myc tag antibody followed by Cy3-labeled anti-mouse antibodies and scFv-hFc using Cy3-labeled anti-human antibodies (red). Nuclei were stained using DAPI (blue). AA2 (a, b), TA10 (c, d), SF9 (e, f) and F2C (g, h) detect respectively Rab6-GTP, Giantin, alpha-Tubulin and nonmuscle MyosinIIA. scFv fused to a hFc portion keep their specificity and are, in general, more sensitive and easier to use than their monomeric scFv counterparts. Bar = 20 μm.

We then assessed the Fc-fused scFvs for detection of antigens by immunofluorescence. After fixation and permeabilization, HeLa cells were incubated with 4 different scFv or with their 4 scFv-hFc counterparts (Figure 2). For each antibody, the detection was at least as good and in general better after Fc fusion, and its usability improved. For example, while the anti-Rab6-GTP scFv scAA2 only gave good labeling when used in conjunction with very short washes [9], human hAA2 produced contrasted labeling using classical immunofluorescence protocols. Similarly, while the scFv TA10 can only detect Giantin when cells are fixed in methanol, the fusion antibody hTA10 can detect Giantin even when cells are fixed by paraformaldehyde (unpublished results). More generally, we systematically saw an increase in the sensitivity of scFv-Fc fusions compared to simple scFvs.

### Multi-species antibodies

**Figure 3:**
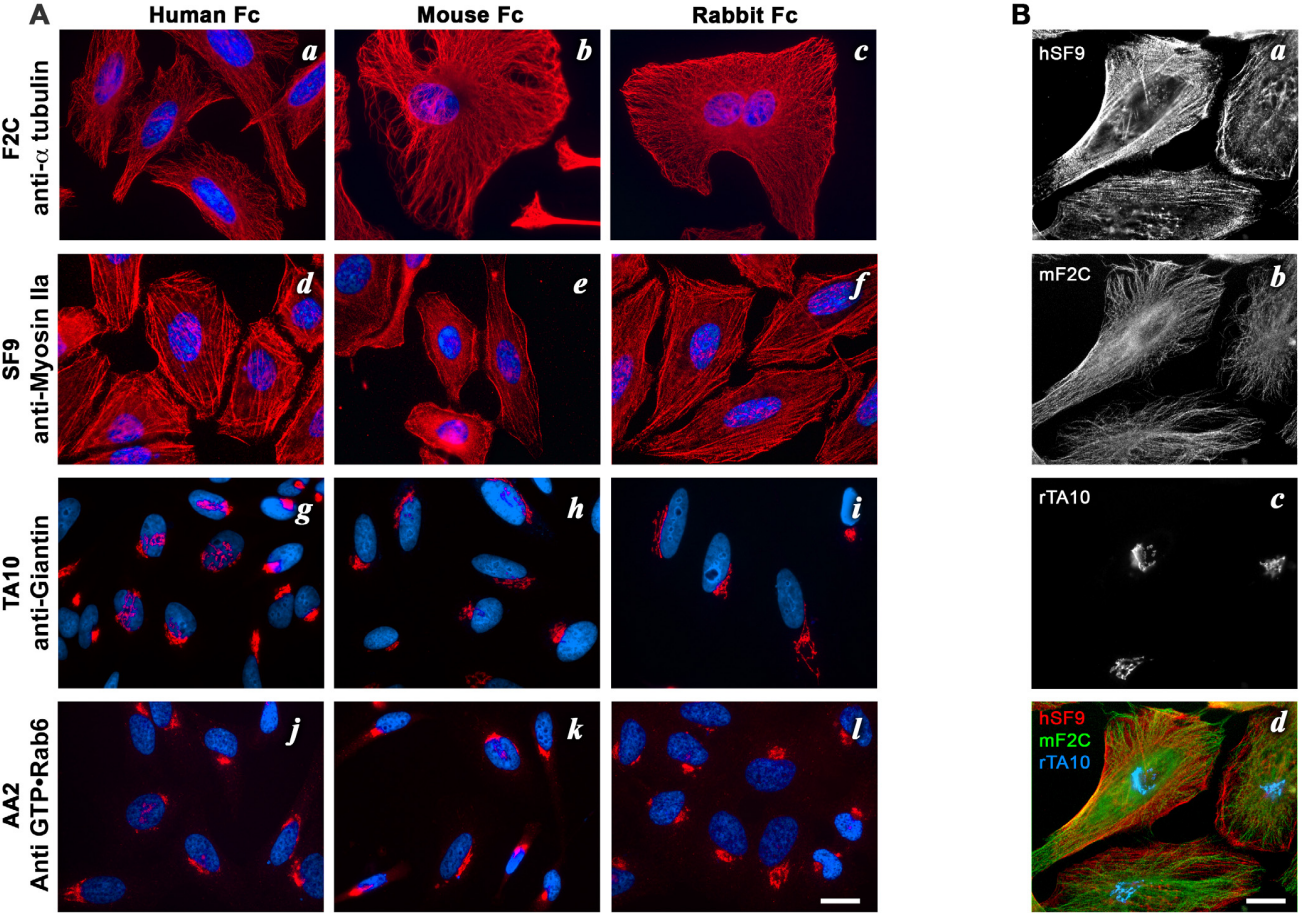
**Immunofluorescence using multi-species antibodies**. A: The scFv-Fc F2C (a-c), SF9 (d-f), TA10 (g-i) and AA2 (j-l) in their human (a, d, g, j), mouse (b, e, h, k) or rabbit (c, f, i, l) versions can be used in immunofluorescence to detect their respective target proteins with similar efficiency (red). Bar = 20 μm. B: Using the multi-species approach, antibodies can be produced fused to any of the three IgG species. This allows a large diversity of multiplexing. In this example, Myosin, Tubulin and Giantin were co-detected in HeLa cells using the human version of SF9 (a), the mouse version of F2C (b) and the rabbit version of TA10 (c) respectively. The three labelings are shown overlayed in (d). Bar = 20 μm.

By fusing scFv to Fc domains, not only are we endowing recombinant antibodies with the same power as natural antibodies for classical immunological methods, but we also generate new and unique tools. Natural antibodies are derived from a single host and thus linked to one species definitively, which complicates multiplexing in many cases. Here, since we developed a series of vectors that allow not only fusion of scFv to human Fc but also to mouse or rabbit IgGs in a single sub-cloning step (see Figure 1), there no longer exists a species barrier in co-immunolabeling applications. AA2, F2C, SF9 and TA10 were introduced in the 3 versions of the pFUSE-Fc modified plasmids and, after transient transfection in CHO cells, scFv-xFc fusion antibodies were recovered in supernatants and used for immunofluorescence assay on HeLa cells (Figure 3). We observed that human, rabbit or mouse versions of each scFv-Fc detected their respective target proteins with similar efficiency (Figure 3A). This thus allows one to carry out multicolor immunofluorescence labeling using any combination of recombinant antibodies (Figure 3B) or of recombinant and natural antibodies (unpublished data).

**Figure 4:**
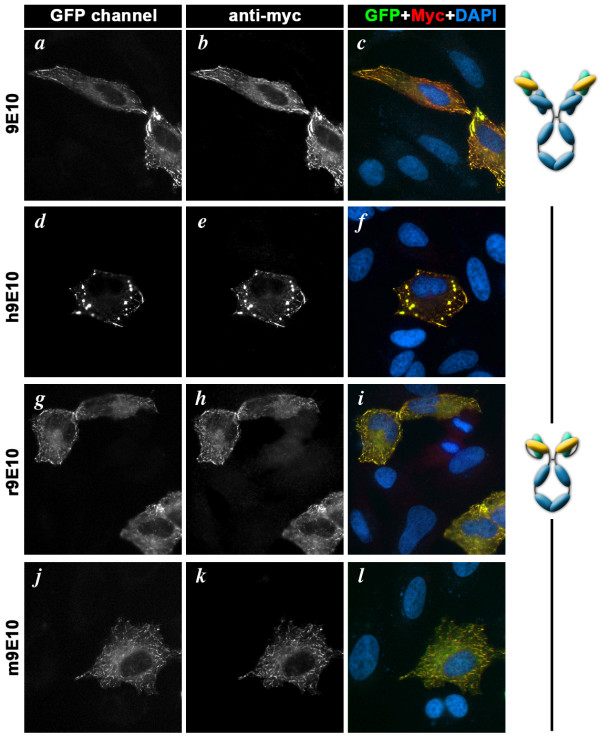
**Immunofluorescence using natural or multi-species anti-Myc 9E10 antibodies**. Hela cells expressing Myc- and GFP-tagged CLIP-170 were immuno-labeled using either the natural mouse monoclonal antibody Myc1-9E10 (a-c) or the recombinant human (d-f), rabbit (g-i) or mouse (j-l) versions of 9E10 scFv fused to Fc domains. 9E10 antibodies were detected using Cy3-labeled secondary antibodies. GFP fluorescence was directly imaged in the green channel (green, a, d, g, h). The overlays (c, f, u, j) show the GFP fluorescence in green and 9E10 detection of Myc-tagged proteins in red. Nuclei, stained using DAPI, are shown in blue. This experiment shows that recombinant 9E10 antibodies efficiently detect Myc tags by immunofluorescence and that the multi-species strategy can be used to change the species of natural antibodies. Bar = 20 μm.

Finally, we showed that multi-species antibody technology can also be adapted to natural antibodies, widening their use. The 9E10 scFv (directed against the widely used Myc tag) has been isolated from Myc1-9E10 hybridoma cells before [10]. We sub-cloned it into our 3 versions of pFUSE-Fc and produced h9E10, r9E10 and m9E10 in CHO cells. The resulting multispecies 9E10 were then tested by immunofluorescence using HeLa cells transiently expressing Myc-tagged GFP-CLIP-170 [11] (Figure 4). This experiment showed that human and rabbit 9E10 were as efficient as the original mouse 9E10 in their ability to detect the overexpressed protein.

## Discussion

With the increasing power of large-sale projects in genomics, transcriptomics and proteomics and the rise of reverse genetics, the need for specific antibodies has dramatically increased [see for example [4,12]]. The recombinant approach is certainly a powerful alternative to classical, animal based approaches, in terms of price, speed and ethics. We and others have demonstrated that a large diversity of antibodies can be obtained this way (e.g. against haptens, proteins, glycolipids, particular conformation or post-translational modifications [2]). However, and despite the identification of high quality recombinant antibodies, their use is still limited, with very few fee-for service recombinant antibody companies on the market and almost no recombinant antibodies in the catalog of antibody providers. This may be due to the fact that, in general, the lack of avidity of monomeric recombinant antibodies strongly limits their sensitivity and that dedicated protocols have to be followed to efficiently use these antibodies. Different approaches have been developed to improve recombinant antibody avidity [see [13]]. The expression system we developed here helps to solve some of the above cited limitations while bringing unique versatility to recombinant antibodies. It additionally allows production of endotoxin-free recombinant antibodies. We have used it so far for more than 12 recombinant antibodies with comparable success (unpublished data). Furthermore, we show that this strategy can be applied not only to synthetic recombinant antibodies but also to natural ones. This may be interesting not only for changing antibody isotype or species but also to save valuable monoclonal antibodies from hybridomas that grow poorly or do not produce anymore. A limitation of our approach however is that some natural antibodies cannot be adapted to the scFv format without a strong loss in affinity.

By fusing scFv to an Fc region, we observed as suggested before [14,15], that the antibodies keep their specificity and gain in sensitivity. scFv-xFc can be used like any natural antibody, without the user actually needing to know that the antibody is a recombinant one. For general use a simple, non purified, cell culture supernatant from CHO cells expressing the antibodies can be diluted between twenty and a thousand times. Importantly, we believe that it is more important to estimate recombinant antibodies "usability" or "effectiveness" instead of their "affinity". This is what is actually done for natural antibodies where the user selects the antibody giving the best signal to noise ratio while the affinity is rarely measured.

A key feature of our strategy is to free the antibody from its species-specificity restriction with the immediate ability, in one single sub-cloning step, to fuse the scFv to different Fc regions belonging to various species. The same antibody and therefore epitope recognized, can thus be used as a human, mouse or rabbit version. This opens up previously unattainable multiplexing possibilities since the antibody species is no longer a factor limiting combinations.

## Conclusion

We developed here a comprehensive and integrated system for multi-species antibody production. It will simplify and strengthen the use of recombinant antibodies for daily laboratory applications and permit a flexibility and choice in multiplexing, previously not feasible. This simple and fully *in vitro *system, with no animal use, will ensure constant and endless production of antibodies belonging to any species and to any isotype. This may represent a breakthrough not only for their general laboratory use but also for diagnosis where multiplexing and constant quality are essential.

## Methods

### Cells and media

HeLa cells were maintained in Dulbecco's Modified Eagle Medium (DMEM) containing 10% heat-inactivated fetal calf serum (FCS), 2 mM L-Glutamine.

Chinese hamster ovary (CHO) cells were maintained in DMEM-F12 containing 10% FCS, 2 mM L-Glutamine.

### Strains and plasmids constructions

The construction of scFv 9E10 (anti-Myc tag) has been described previously [10].

The NotI site (4025) present in the pFUSE-hFc2(IL2ss) vector (InvivoGen, CA, USA) was mutated by fill-in using the Klenow enzyme (Biolabs) followed by auto-ligation. Then a multiple cloning site (MCS) was added between the IL2ss and the hIgG2Fc by ligating a synthetic adaptor (5' AATTC GATAT CGGCC ATGGT TTGGT ACCTT GC 3' and 5' GATCT AGCGG CCGCA AGGTA CCAAA CCATG GC 3') between EcoRI (626) and BglII(648). The final construct contains the following MCS : EcoRI-EcoRV-NcoI-NotI-BglII.

pFUSE-rFc2(IL2ss) and pFUSE-mFc2(IL2ss) vectors were mutated in their Fc part (respectively IgG and IgG2a) to remove the NcoI site using a QuikChange site-directed mutagenesis kit (Stratagene, CA, USA) and the following primers : for rabbit Fc : forward primer 5' AGCCG AAGGT CTACA CAATG GGCCC TCCCC GGGA 3' and backward primer 5' TCCCG GGGAG GGCCC ATTGT GTAGA CCTTC GGCT 3' ; for mouse Fc : forward primer 5' TGATC TCCCT GAGCC CTATG GTCAC ATGTG TGGT 3' and backward primer 5' ACCAC ACATG TGACC ATAGG GCTCA GGGAG ATCA 3'

The Fc fragment of rabbit or mouse vectors were then sub-cloned into the mutated pFUSE-hFc2(IL2ss) vector using BglII and NheI. scFvs were then inserted in the modified vectors between NcoI and NotI. The plasmids were deposited on Genbank under accession numbers FJ716123, FJ716124 and FJ716125.

### Transient transfection

CHO cells were cultivated into 75 cm^2 ^tissue culture flasks. Twenty four or forty eight hours after, when the cells reached 90% confluence, transfection was performed using 20 μg of plasmid DNA and 30 μL of lipofectamine2000 (Invitrogen, La Jolla, USA) during 4h30 according to the manufacturer's recommendations. Transfection medium was then removed and cells were incubated at 30°C with fresh DMEM-F12 for 72–96 hours. Cell supernatants were then harvested and kept at 4°C, or aliquoted and stored at -20°C. Stable cell lines were also selected for some antibodies. Depending on the antibody expressed, we obtained between 0.2 and 2 mg of antibody per Liter of medium. It is likely however that higher production may be achievable after optimization [see [16]].

### Western Blot

After boiling in SDS-PAGE loading buffer, the samples were separated on a 12% SDS-PAGE and transferred to nitrocellulose membranes (Whatman GmbH, Dassel, Germany). For the experiment presented in Figure 1C, a loading buffer that did not contain DTT was used. Membranes were blocked in 3% non-fat milk-PBS (Phosphate Buffer Saline) with 0.2% Tween 20 for 1 h at room temperature or overnight at 4°C. Supernatants of scFv-xFc were used at 1/300 and added to the membranes for 1 h. Blots were then washed and incubated 1 h with secondary anti-human (or anti-mouse or anti-rabbit) HRP labeled antibodies (diluted at 1/10000 in PBS 0.1% Tween 20) (Jakson ImmunoResearch Laboratories, Westgrove, PA, USA). After 5 washes with PBS 0.1% Tween 20, secondary antibodies were then revealed using the SuperSignal chemoluminescent reagent (Pierce, Rockford, IL, USA) and Hyperfilm ECL (GE HealthCare, Velizy, France).

### Immunofluorescence

Immunofluorescence labeling were performed on HeLa cells. Cells were either fixed in 3% paraformaldehyde and permeabilized with PBS plus 0.1% saponin or fixed and permeabilized with cold methanol for 4 min at -20°C. scFv-xFc-containing supernatants were used diluted from 1/20 (anti-Myc) to 1/1000 (anti-Giantin) times and incubated for 1 hr on cells. Cells were then rinsed twice and incubated with secondary antibodies for 30 min (Jackson ImmunoResearch Laboratories, Westgrove, PA, USA). Purified scFv were used as described using short washes [9].

## Authors' contributions

SM constructed the plasmids, expressed the antibodies, set-up immunolabeling conditions and help to draft the manuscript. AEM was involved in setting-up large scale antibody production. OV participated in plasmid construction and in setting-up antibody production and use. CN was involved in the initial steps of development. PB contributed to setting-up the immunolabeling. SD provided the 9E10 scFv. FP conceived of the study, coordinated it and helped to draft the manuscript.
